# Supplementary material for: A machine learning one-class logistic regression model to predict stemness for single cell transcriptomics and spatial omics
Source: BMC Genomics. 2023 Nov 28;24:717. doi: 10.1186/s12864-023-09722-6 (PMC10683105; doi:10.1186/s12864-023-09722-6)
Supplement: Supplementary file 1 — Supplemental Figure 1. (A) Stemness on bootstrapped high and poor quality breast cancer samples on dataset 1 (i,ii) and dataset 2 (iii,iv). Each figure represents one sample and the box plot indicates the stemness distribution of a subset of cells that grows in number to the right untill the total cells for that given sample. (B) Rank barplot of number of cells by patient samples for dataset 1 (i) and dataset 2 (ii). Supplemental Figure 2. Gene expression analysis of stemness model. Volcano plots of top stemness A) cancer cycling cells from dataset 1 (i) and dataset 2 (ii) respectively. Gradient depicts negative logFC (blue) to positive logFC (red). Y axis represents the statistical significance and X axis the different percentage of cells expressing a given gene. Positive different percentage means that top stemnes cells have more cells expressing that gene. B) GO enrichment analysis of the top regulated genes in cancer cycling cells for dataset 1 (red) and dataset 2 (blue). The number of DEG is plotted on the X axis and GO categories the genes are represented in are plotted on the y axis. C) Volcano plots of top stemness cycling T cells from dataset 1 (i) and dataset 2 (ii) respectively. Gradient depicts negative logFC (blue) to positive logFC (red). D) GO enrichment analysis comparison from the number of up regulated genes in cycling t-cells dataset 1 (red) and dataset 2 (blue). E) Gene similarity score of upregulated genes from cancer basal high stemness cells vs cluster markers genes. On the left, dataset 1 high stemness genes are compared to gene markers from dataset 2. On the right, dataset 2 high stemness genes are compared to gene markers from dataset 1. Gradient depicts less gene similarity (blue) to greater gene similarity (red). Supplemental Figure 3. (A) Heatmap of gene similarity analysis of cluster markers in dataset 1 compared to dataset 2 for cancer basal DEGs and (B) heatmap of gene similarity analysis of cluster markers in da [file 12864_2023_9722_MOESM1_ESM.pdf]

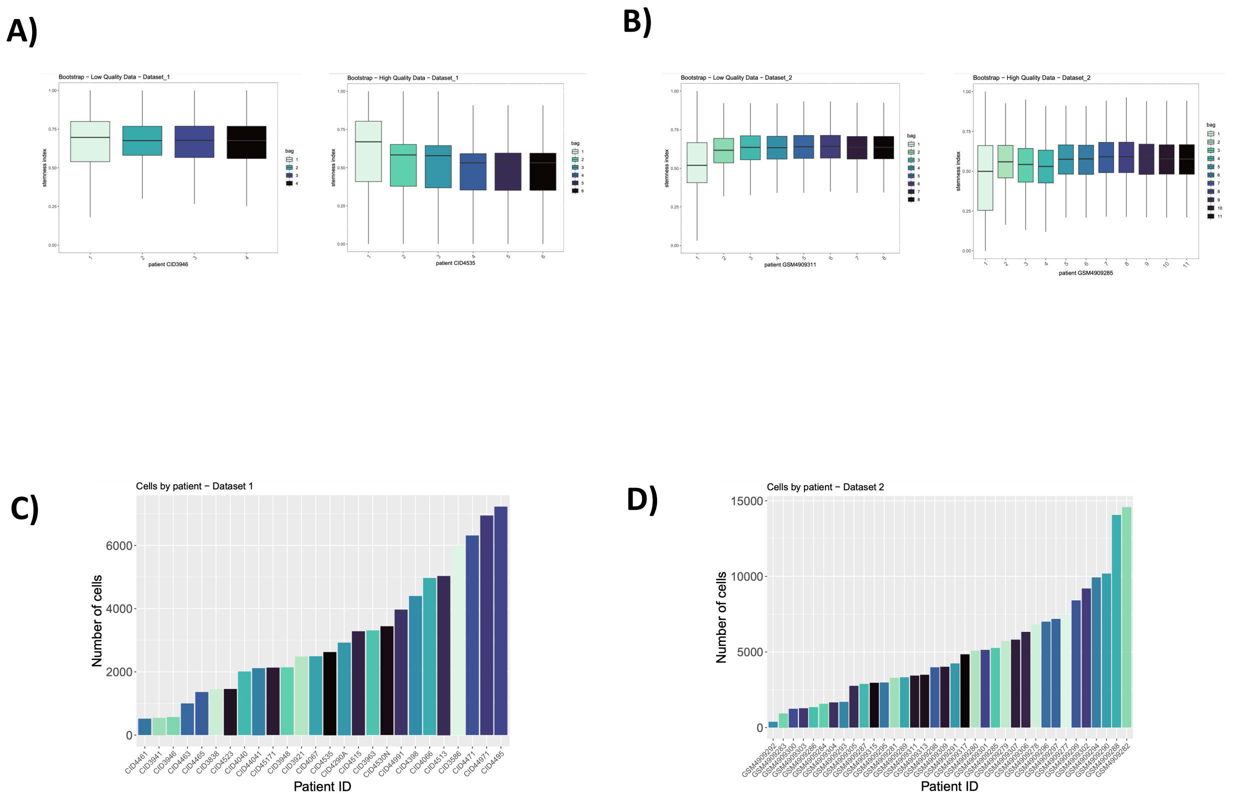

Supplemental Figure 1 (A) Stemness on bootstrapped high and poor quality breast cancer samples on dataset 1 (i,ii) and dataset 2 (iii,iv). Each figure represents one sample and the box plot indicates the stemness distribution of a subset of cells that grows in number to the right until the total cells for that given sample. (B) Rank barplot of number of cells by patient samples for dataset 1(i) and dataset 2 (ii).

Supp. Figure 1

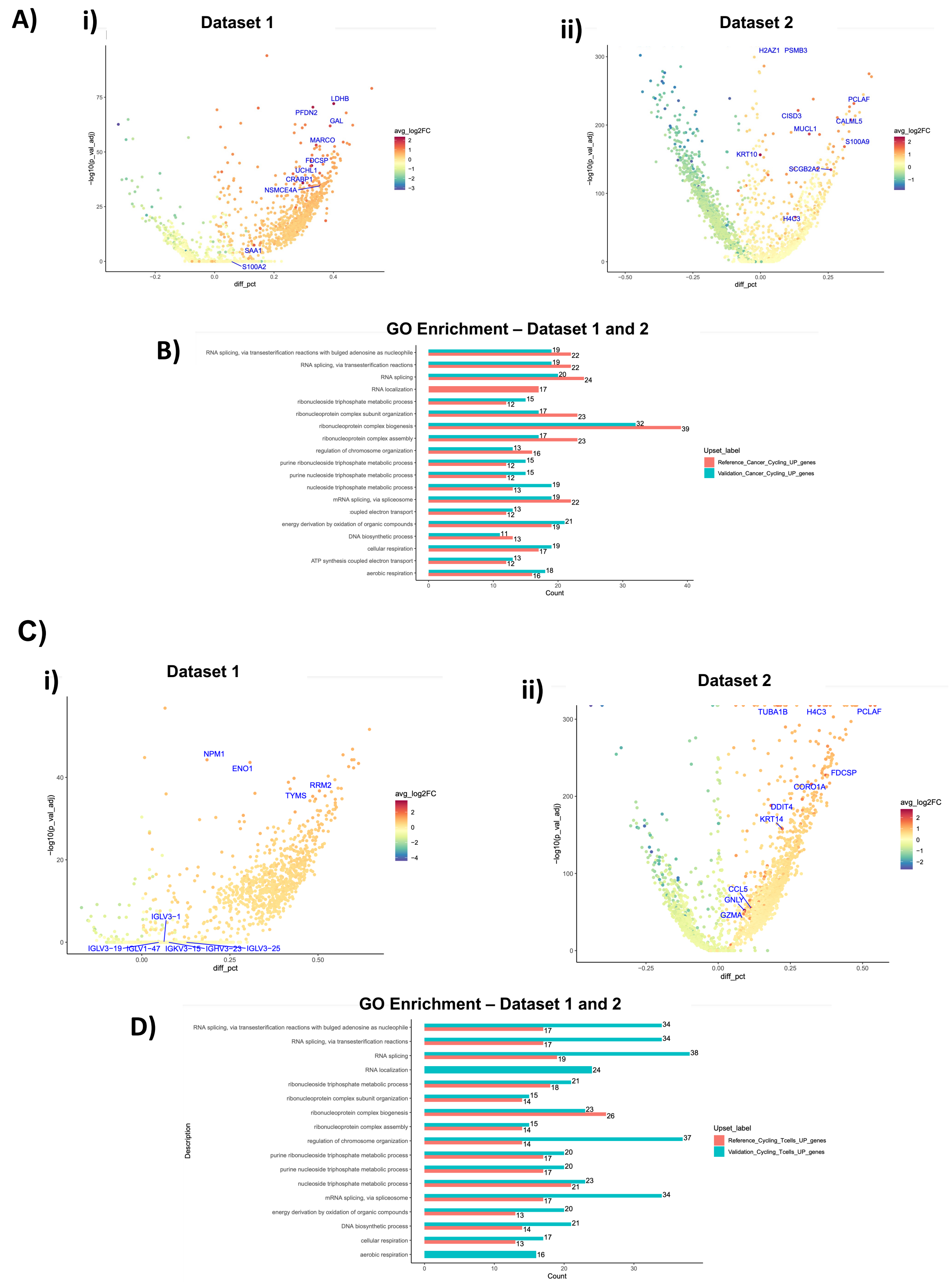

Supp. Figure 2

Supplemental Figure 2 Gene expression analysis of stemness model. Volcano plots of top stemness A) cancer cycling cells from dataset1 (i) and dataset 2 (ii) respectively. Gradient depicts negative logFC (blue) to positive logFC (red). Y axis represents the statistical significance and X axis the different percentage of cells expressing a given gene. Positive different percentage means that top stemness cells have more cells expressing that gene. B) GO enrichment analysis of the top regulated genes in cancer cycling cells for dataset 1 (red) and dataset 2 (blue). The number of DEG is plotted on the X axis and GO categories the genes are represented in are plotted on the y axis. C) Volcano plots of top stemness cycling T cells from dataset1 (i) and dataset 2 (ii) respectively. Gradient depicts negative logFC (blue) to positive logFC (red). D) GO enrichment analysis comparison from the number of up regulated genes in cycling t-cells dataset 1 (red) and dataset 2 (blue). E) Gene similarity score of upregulated genes from cancer basal high stemness cells vs cluster markers genes. On the left, dataset 1 high stemness genes are compared to gene markers from dataset 2. On the right, dataset 2 high stemness genes are compared to gene markers from dataset 1. Gradient depicts less gene similarity (blue) to greater gene similarity (red).

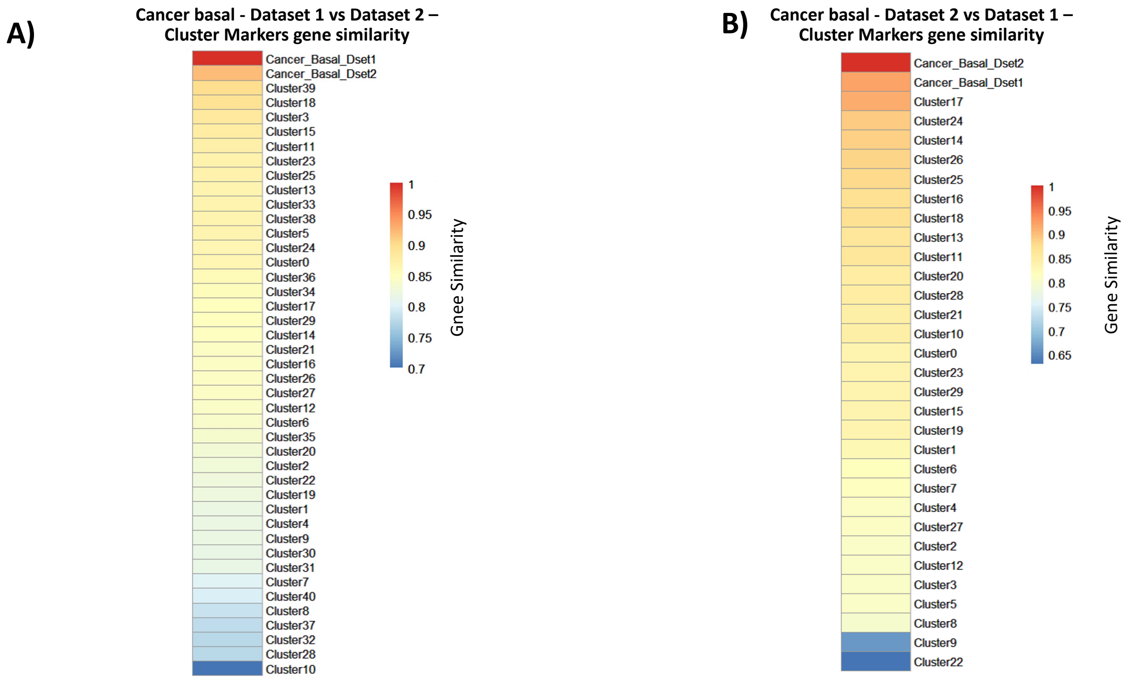

Supplemental Figure 3 (A) Heatmap of gene similarity analysis of cluster markers in dataset 1 compared to dataset 2 for cancer basal DEGs and (B) heatmap of gene similarity analysis of cluster markers in dataset 2 compared to dataset 1 for the same cell type.

Supp. Figure 3

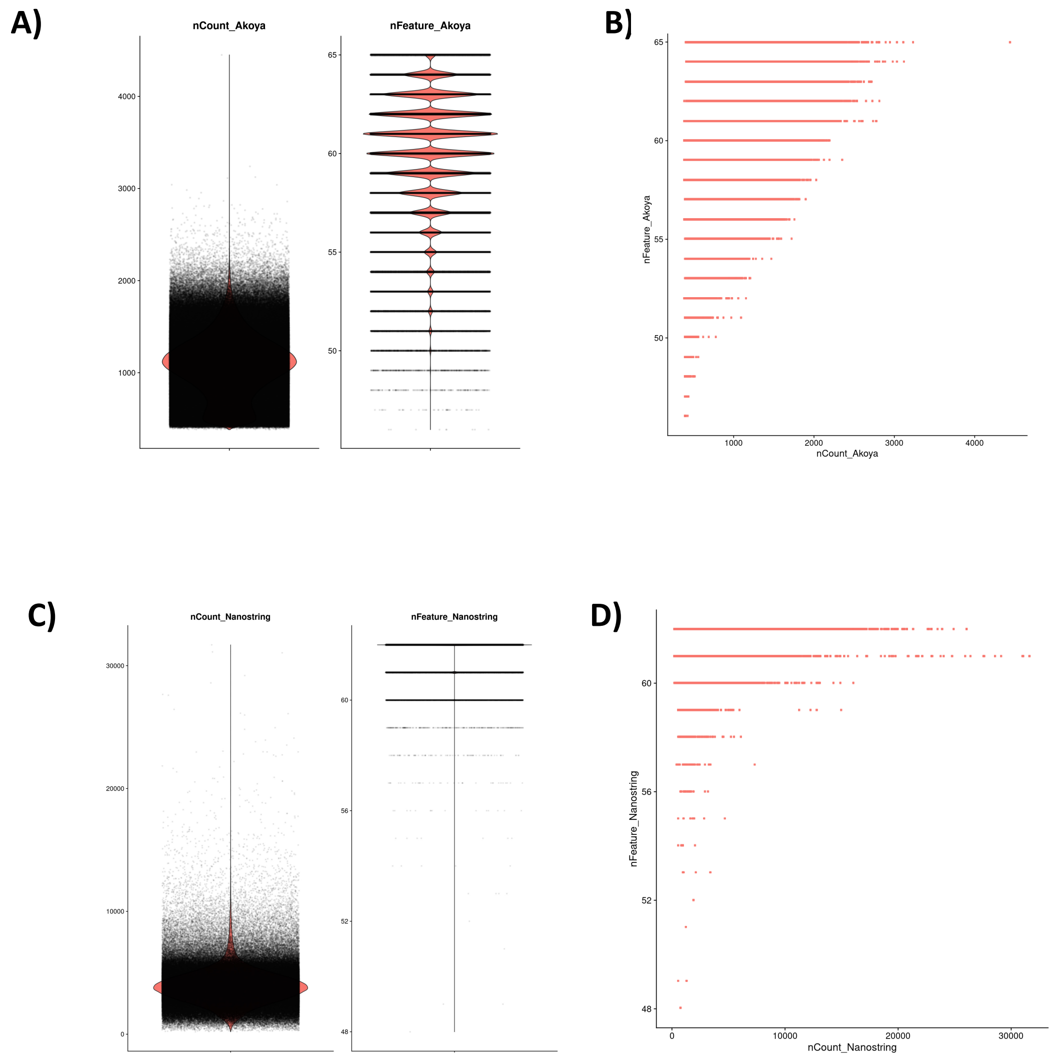

Supplemental Figure 4 QC Metrics of Akoya PhenoCycler and Nanostring CosMx samples. A) Violin plot of fluorescence intensity signal (nCount_Akoya) showing the number of proteins detected per cell (nFeature_Akoya). B) Scatter plot of number of fluorescence intensity (x-axis)

Supp. Figure 4
